# T1rho and T2 relaxation times of the normal adult knee meniscus at 3T: analysis of zonal differences

**DOI:** 10.1186/s12891-017-1560-y

**Published:** 2017-05-18

**Authors:** Shoichiro Takao, Tan B. Nguyen, Hon J. Yu, Shigeo Hagiwara, Yasuhito Kaneko, Taiki Nozaki, Seiji Iwamoto, Maki Otomo, Ran Schwarzkopf, Hiroshi Yoshioka

**Affiliations:** 1 Department of Radiological Sciences, University of California, Irvine, 101 The City Drive South, Rt. 140, Orange, CA 92868 USA; 2 Department of Radiology, Tokushima University Hospital, 3-18-15, Kuramoto-Cho, Tokushima City, 770-8509 Tokushima Japan; 3 Department of Orthopaedic Surgery, University of California, Irvine, 101 The City Drive South, Orange, 92868 CA USA; 4 Department of Diagnostic Radiology, Graduate School of Health Sciences, Tokushima University, 3-18-15, Kuramoto-Cho, Tokushima City, 770-8509 Tokushima Japan

**Keywords:** Magnetic resonance imaging, T1rho, T2 mapping, Knee, Meniscus, Zonal differentiation

## Abstract

**Background:**

Prior studies describe histological and immunohistochemical differences in collagen and proteoglycan content in different meniscal zones. The aim of this study is to evaluate horizontal and vertical zonal differentiation of T1rho and T2 relaxation times of the entire meniscus from volunteers without symptom and imaging abnormality.

**Methods:**

Twenty volunteers age between 19 and 38 who have no knee-related clinical symptoms, and no history of prior knee surgeries were enrolled in this study. Two T1rho mapping (b-FFE T1rho and SPGR T1rho) and T2 mapping images were acquired with a 3.0-T MR scanner. Each meniscus was divided manually into superficial and deep zones for horizontal zonal analysis. The anterior and posterior horns of each meniscus were divided manually into white, red-white and red zones for vertical zonal analysis. Zonal differences of average relaxation times among each zone, and both inter- and intra-observer reproducibility were statistically analyzed.

**Results:**

In horizontal zonal analysis, T1rho relaxation times of the superficial zone tended to be higher than those of the deep zone, and this difference was statistically significant in the medial meniscal segments (84.3 ms vs 76.0 ms on b-FFE, *p* < 0.0001 and 96.5 ms vs 91.7 ms on SPGR, *p* = 0.004). In vertical zonal analysis, T1rho relaxation times of the white zone tended to be higher than those of the red zone, and this difference was statistically significant in the posterior horn of the medical meniscus (88.4 ms vs 77.1 ms on b-FFE, *p* < 0.001 and 104.9 ms vs 96.8 ms on SPGR, *p* =0.001). Likewise, T2 relaxation times of the superficial zone were significantly higher than those of the deep zone (80.4 ms vs 74.4 ms in the medial meniscus, *p* = 0.011). T2 relaxation times of the white zone were significantly higher than those of the red zone in the medial meniscus posterior horn (96.8 ms vs 84.3 ms, *p* < 0.001) and lateral meniscus anterior horn (104.6 ms vs 84.2 ms, *p* < 0.0001). Inter-class and intra-class correlation coefficients were excellent (>0.74) or good (0.60–0.74) in all meniscal segments on both horizontal and vertical zonal analysis, except for inter-class correlation coefficients of the lateral meniscus on SPGR. Compared with SPGR T1rho images, b-FFE T1rho images demonstrated more significant zonal differentiation with higher inter- and intra-observer reproducibility.

**Conclusions:**

There are zonal differences in T1rho and T2 relaxation times of the normal meniscus.

## Background

The meniscus of the knee joint serves important functions for shock absorption, joint stability and joint lubrication [1–3]. Patients with meniscus injury or degeneration may develop osteoarthritis of the knee [4].

The extracellular matrix of normal meniscus is composed of 72% water, 22% collagen and 0.8% glycosaminoglycans [5]. This composition may change with degeneration [5]. Recently, with advances in imaging software and magnetic resonance imaging (MRI) sequences, it is believed that quantitative analysis of T1rho (T1 relaxation time in the rotating frame) and T2 relaxation times of articular cartilage and meniscus can be examined to reveal the underlying composition of extracellular matrix. Previous studies have shown usefulness of quantitative evaluation using MR relaxation times of the knee meniscus [6–15]. However, few studies about zonal differentiation of normal meniscal T1rho and T2 relaxation times have been published [7, 12, 15]. Previous studies have evaluated vertical differences in MR relaxation times with comparisons of the inner one-third, middle one-third and outer one-third. However, more detailed analysis including evaluation of horizontal zonal differences between superficial and deep zones has not been performed. Therefore, the purpose of this study is to evaluate vertical and horizontal zonal differentiation of T1rho and T2 relaxation times of the entire meniscus from volunteers without symptom and imaging abnormality. We also investigate inter-observer and intra-observer reproducibility of meniscus segmentation in this study.

## Methods

### Subjects

Twenty-three volunteers were recruited for this study. Inclusion criteria for all subjects were: age between 18 and 40 years, no knee-related clinical symptoms and no history of prior knee surgeries. One subject was excluded from the study because of a large knee that was unable to fit within the knee coil, and two subjects were excluded due to claustrophobia in the MRI scanner. A total of 20 subjects (male : female = 13 : 7, right-knee : left-knee = 10 : 10) were included in the study. Mean age was 29 years (range: 19–38) and mean body weight was 73 kg (range: 50–100). The study protocol was approved by the Institutional Review Board at the University of California, Irvine and conformed to the Declaration of Helsinki. Written informed consent was obtained from each subject.

### MR imaging protocol

All MR images were acquired on a 3.0-Tesla scanner (Achieva TX, Philips Healthcare, Best, Netherland) using an 8-channel receive-only knee RF coil. For each subject, two sagittal T1rho relaxation time sequences (b-FFE [balanced-fast field echo] and SPGR [spoiled gradient-recalled acquisition]) and one sagittal T2 relaxation time sequence were acquired. MR imaging acquisition parameters were as follows (Table 1) : b-FFE T1rho: mode = 3D, repetition time (TR)/echo time (TE) = 4.8/2.4 ms, bandwidth = 606 Hz, flip angle (FA) = 25°, echo train length (ETL) = 154, fat-saturation method = SPIR (spectral presaturation with inversion recovery), frequency of spin-lock = 575 Hz, time of spin-lock (TSL) = 20/40/60/80 ms, acceleration factor = 2, acquisition time = 15 min 48 s. SPGR T1rho: mode = 3D, TR/TE = 6.4/3.4 ms, bandwidth = 475 Hz, FA = 10°, ETL = 64, fat-saturation method = PROSET (principle of selective excitation technique), frequency of spin-lock = 575 Hz, TSL = 20/40/60/80 ms, acceleration factor = 2, acquisition time = 16 min 36 s. T2 mapping: mode = 2D, TR = 2700 ms, TE = 13/26/39/52/65/78/91 ms, bandwidth = 243 Hz, FA = 90°, ETL = 7, acceleration factor = 2.2, acquisition time = 13 min 25 s. All images were obtained with FOV = 140 × 140 mm, image matrix = 512 × 512, slice thickness/slice gap = 3/0 mm, number of slices = 31.

**Table 1:** MR imaging parameters

Imaging parameters	b-FFE T1rho	SPGR T1rho	T2
Mode	3D	3D	2D
Sequence	balanced FFE	SPGR	TSE
Plane	sagittal	sagittal	sagittal
Fat sat	SPIR	ProSet	No
Matrix	304 × 302	388 × 252	388 × 319
Number of slices	31	31	31
FOV (mm)	140 × 140	140 × 140	140 × 140
Slice thickness (mm)	3.0	3.0	3.0
Slice gap (mm)	0.0	0.0	0.0
Flip angle	25	10	90
TE (ms)	2.4	3.4	13/26/39/52/65/78/91 (7-TE)
TR (ms)	4.8	6.4	2700
BW (Hz/pixel)	606	475	243
Echo train length	154	64	7
NEX	1	1	1
Effective inplane spatial resolution (mm)	0.27 × 0.27	0.27 × 0.27	0.27 × 0.27
Acceleration factor	2	2	2.2
Spin-lock frequency (Hz)	575	575	N.A.
Time of spin-lock (TSL) (ms)	20/40/60/80	20/40/60/80	N.A.
Acquisition time	3:57 × 4	4:09 × 4	13:25

Abbreviations; *FFE* fast field echo, *SPGR* spoiled gradient-recalled acquisition, *SPIR* spectral presaturation with inversion recovery, *ProSet* principle of selective excitation technique, *FOV* field of view, *TE* echo time, *TR* repetition time, *BW* bandwidth, *NEX* number of excitations

In addition to T1rho and T2 mapping sequences, turbo spin echo 2D fat suppressed sagittal proton density weighted images (TSE FS PDWI) were acquired for evaluation of morphological and signal abnormality of the knee menisci. MR imaging acquisition parameters were as follows : mode = 2D, TR/TE = 4300/30 ms, bandwidth = 168 Hz, FA = 90°, ETL = 15, fat-saturation method = SPAIR (spectral attenuated inversion recovery), FOV = 140 × 140 mm, image matrix = 512 × 512, slice thickness/slice gap = 3/0 mm, number of slices = 3, acceleration factor = 1.1, acquisition time = 3 min 35 s. S.T. (10 years experience in musculoskeletal radiology) read the sagittal TSE FS PDWI MR images to ascertain no morphological nor signal abnormalities was present in all menisci.

### Meniscus segmentation and image processing

Segmentation of the meniscus was performed using MIPAV (Medical Image Processing, Analysis and Visualization) version 7.2.0 software. The entire meniscus was segmented manually slice-by-slice by drawing polygon regions of interest (ROIs), with manual exclusion of adjacent articular cartilage, joint effusion and soft tissue structures. For b-FFE T1rho and SPGR T1rho mapping, images with TSL = 20 ms were chosen for segmentation. For T2 mapping, images with TE = 13 ms were chosen for segmentation. Lateral meniscus (LM) and medial meniscus (MM) were divided into anterior horn (LMAH, MMAH), posterior horn (LMPH, MMPH) and body (LMBD, MMBD) segments, respectively. Meniscal body was defined as the section where the anterior and posterior portions of the meniscus were connected. Meniscal root attachments were eliminated from this study. Segmentation of the meniscus was independently performed by a board-certified radiologist with subspecialization in musculoskeletal radiology (R1, 10 years experience in musculoskeletal radiology) and a radiology resident physician (R2, 3 years experience in radiology residency).

Images were transferred in DICOM (Digital Imaging and Communications in Medicine) format to a personal computer (PC; Windows 7), which was used to perform all post-processing and analysis. To correct for possible motion between the scans, the T1rho and T2 sequences were first coregistered with respect to the first TSL/TE images using rigid-body transformation before being fitted to a monoexponential function on a pixel-by-pixel basis for generation of T1rho maps: S (TSL) = S0 * exp (−TSL/T1rho) and T2 maps: S (TE) = S0 * exp (−TE/T2), where S0 is the signal intensity when TSL/TE = 0. The meniscus was then extracted from the first TSL/TE images on a slice-by-slice basis.

All of image processing described above was performed using software in Matlab (Mathworks, Natick, MA) that was developed and implemented in-house.

T1rho and T2 relaxation times of each ROI were plotted pixel-by-pixel to spreadsheets in Microsoft Excel.

### Zonal division of the meniscus

**Fig. 1:**
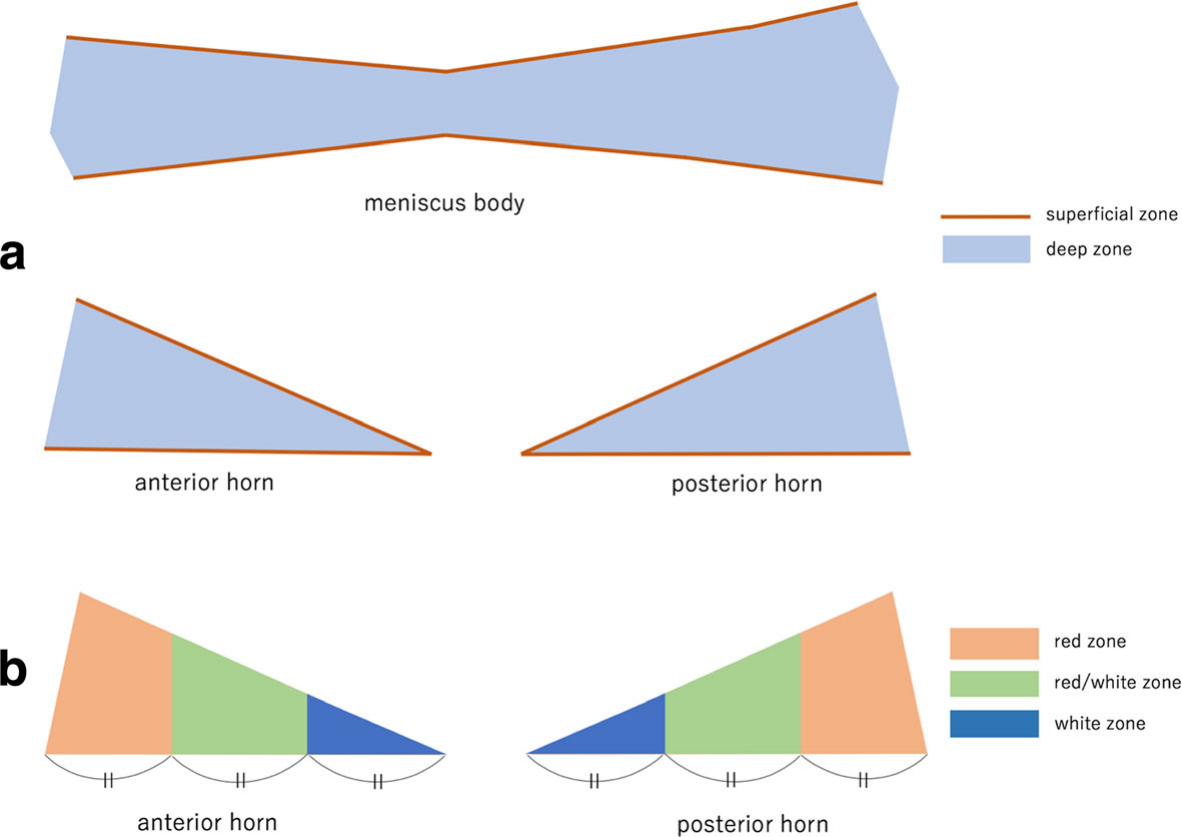
Zonal analysis of the meniscus. **a** In horizontal zonal analysis, superficial zone (*brown*) is the first layer of pixels of ROI along meniscal surface. Deep zone (*blue*) is the reminder of the pixels of ROI. **b** In vertical zonal analysis, white zone (*deep blue*) is the inner 1/3, red/white zone (*green*) is the middle 1/3, and red zone (*orange*) is outer 1/3 of meniscus

Each meniscus was divided manually into superficial (the first layer of pixels of the ROI along the meniscal surface) and deep (the remainder of the pixels of the ROI) zones for horizontal zonal analysis (Fig. 1a). This horizontal zonal analysis was applied to the entire meniscus.

In vertical zonal analysis, the anterior and posterior horns of the meniscus were divided manually into three zones from the free edge to the periphery of the meniscus: white zone (inner 1/3), red-white zone (middle 1/3) and red zone (outer 1/3) (Fig. 1b). This vertical zonal analysis was not applied to the meniscal body.

### Analysis of the T1rho and T2 values

Average relaxation time was calculated and compared between superficial and deep zones for horizontal zonal analysis. This comparison was performed on LMAH, LMPH, LMBD, MMAH, MMPH and MMBD. Average relaxation time was also compared between red, red-white and white zones for vertical zonal analysis. This comparison was performed on LMAH, LMPH, MMAH and MMPH. Pixels with relaxation time of 0 were excluded due to misregistration or inadequate fitting.

### Statistical analysis

Average relaxation times of each zone were calculated and compared using the Mann–Whitney test. For analysis of the inter-observer and intra-observer reproducibility of the meniscus segmentation (pixel counts in each ROI), we used Bland-Altman plot and calculated inter-class and intra-class correlation coefficients (inter-ICC and intra-ICC). For analysis of the inter-observer and intra-observer reproducibility of the relaxation times, we calculated inter-ICC and intra-ICC. On this study, the relative strength of agreement is defined as follows: poor (ICC: less than 0.40), fair (ICC: 0.40–0.59), good (ICC: 0.60–0.74) and excellent (ICC: greater than 0.74) [16]. Statistical analyses were performed using MedCalc for Windows, version 15.8 (MedCalc Software, Ostend, Belgium).

## Results

### Zonal analysis of average T1rho and T2 relaxation times of the meniscus

**Fig. 2:**
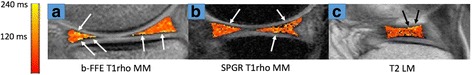
Representative color maps demonstrate T1rho (**a** and **b**) and T2 (**c**) relaxation times are higher in superficial zones than deep zones in the horizontal zonal analysis. These images are from different subjects

Table 2 shows the results of horizontal zonal analysis. T1rho relaxation times of superficial zone were significantly higher than those of deep zone in all analyzed segments on b-FFE except LMBD and in MMPH on SPGR. Likewise, T2 relaxation times of superficial zone were significantly higher than those of deep zone in LMAH, MMBD and MMPH. Representative relaxation time color maps of the meniscus for horizontal zonal analysis are shown in Fig. 2.

**Table 2:**
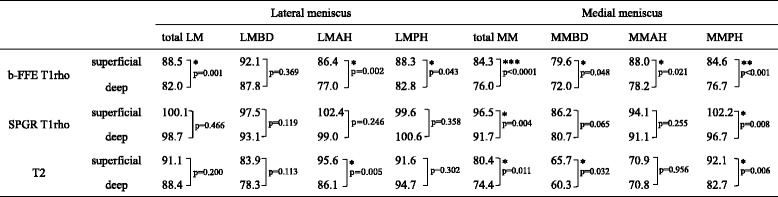
Comparison of average T1rho and T2 relaxation times in horizontal zones

(^***^ *p* < 0.0001, ^**^ *p* < 0.001, ^*^ *p* < 0.05)

**Fig. 3:**
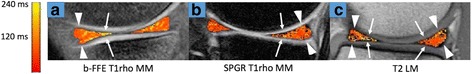
Representative color maps demonstrated T1rho relaxation times of white zone (*arrows*) are higher than red zone (*arrow heads*) in MMAH (**a**) and MMPH (**b**) in the vertical zonal analysis. T2 relaxation time of the white zone is higher than red zone in LMAH, but lower in LMPH (**c**). These images are from different subjects

Table 3 shows the results of vertical zonal analysis. T1rho relaxation times of white zone were significantly higher than those of red zone in all segments except LMPH on b-FFE and only MMPH on SPGR. T2 relaxation times in vertical analysis showed mixed results. T2 relaxation times of white zone were significantly higher than those of red zone in LMAH and MMPH, but significantly lower in LMPH. Representative relaxation time color maps of the meniscus for vertical zonal analysis are shown in Fig. 3.

**Table 3:**
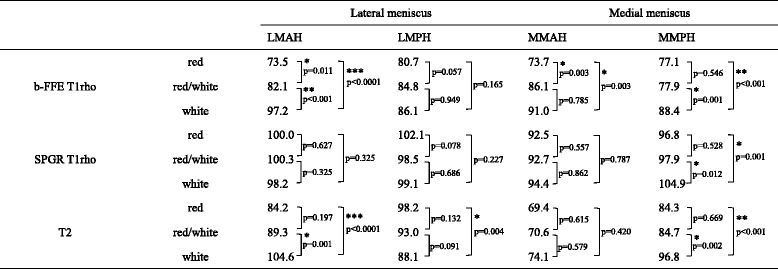
Comparison of average T1rho and T2 relaxation times in vertical zones

(^***^ *p* < 0.0001, ^**^ *p* < 0.001, ^*^ *p* < 0.05)

### Inter- and intra-observer reproducibility of manual segmentation among b-FFE T1rho, SPGR T1rho and T2 relaxation sequences (pixel counts in each ROI)

**Fig. 4:**
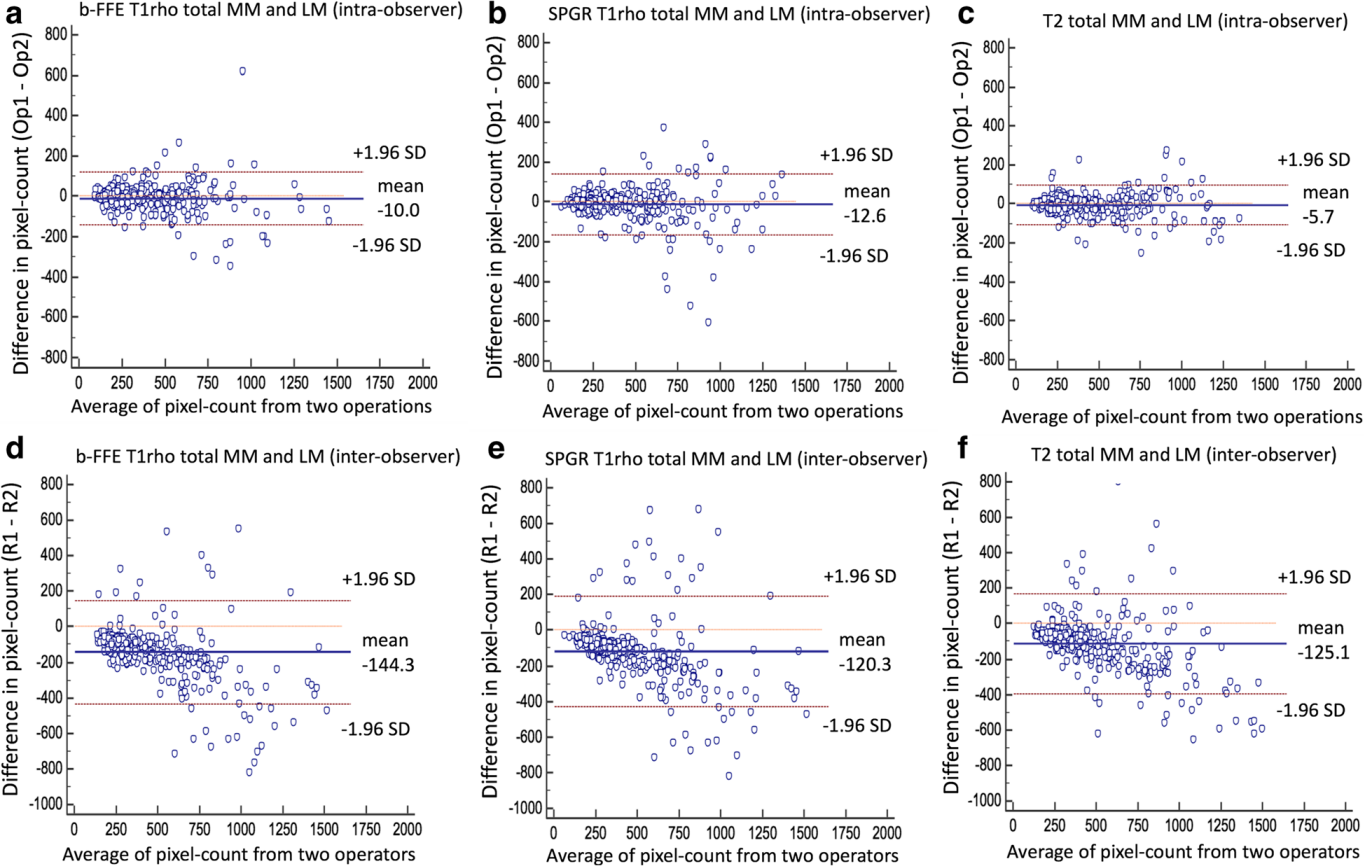
Bland-Altman plots of pixel counts in meniscus segmentation. **a** differential plot of intra-observer agreement on b-FFE T1rho. **b** differential plot of intra-observer agreement on SPGR T1rho. **c** differential plot of intra-observer agreement on T2. **d** differential plot of inter-observer agreement on b-FFE T1rho. **e** differential plot of inter-observer agreement on SPGR T1rho. **f** differential plot of inter-observer agreement on T2

Intra- and inter-ICC of pixel counts in each ROI are shown in Table 4. Intra- and inter-ICC of total LM and total MM were excellent among all sequences. However, in the analysis of each segment, inter-ICC of LMBD resulted in poor to fair reproducibility (0.51 on b-FFE, 0.35 on SPGR and 0.49 on T2). Bland-Altman plots of pixel counts are shown in Fig. 4.

**Table 4:** Intra- and inter-class correlation coefficient (intra- and inter-ICC) in pixel counts in meniscus segmentation

		Lateral meniscus				Medial meniscus			
		total LM	LMBD	LMAH	LMPH	total MM	MMBD	MMAH	MMPH
b-FFE T1rho	Intra-ICC	0.97	0.90	0.90	0.86	0.95	0.85	0.87	0.97
	Inter-ICC	0.85	0.51	0.66	0.89	0.85	0.59	0.83	0.88
SPGR T1rho	Intra-ICC	0.94	0.77	0.90	0.88	0.95	0.85	0.93	0.93
	Inter-ICC	0.80	0.35	0.76	0.87	0.90	0.71	0.82	0.90
T2	Intra-ICC	0.98	0.90	0.92	0.89	0.98	0.94	0.92	0.98
	Inter-ICC	0.86	0.49	0.63	0.67	0.88	0.70	0.82	0.88

### Inter- and intra-observer reproducibility of manual segmentation among b-FFE T1rho, SPGR T1rho and T2 relaxation sequences (relaxation times)

Table 5 demonstrates intra- and inter-ICC of relaxation time of each ROI in horizontal zonal analysis. Intra-ICC of T1rho and T2 from total LM and MM were excellent. In the analysis of each segment, intra-ICC of the medial and lateral meniscal segments on b-FFE were excellent (0.90–0.95), while those on SPGR were lower than on b-FFE and were good to excellent (0.70–0.81 in the lateral meniscus and 0.83–0.89 in the medial meniscus). Intra-ICC of each segment on T2 were all excellent (0.92–0.96). Inter-ICC from total LM and MM were excellent on b-FFE, fair on SPGR and good on T2. Inter-ICC in the segmental analysis were fair to excellent (0.58–0.89) on b-FFE, fair to excellent (0.41–0.79) on SPGR, and fair to excellent (0.58–0.75) on T2. On T1rho, inter-ICC of the MM was higher than those of the LM, and inter-ICC on b-FFE was higher than those on SPGR. T2 showed similar inter-ICC between the medial and lateral menisci.

**Table 5:** Intra- and inter-class correlation coefficient (intra- and inter-ICC) in horizontal zonal analysis

		total LM and MM	Lateral meniscus				Medial meniscus			
			total LM	LMBD	LMAH	LMPH	total MM	MMBD	MMAH	MMPH
b-FFE T1rho	Intra-ICC	0.93	0.93	0.94	0.95	0.90	0.94	0.95	0.91	0.95
	Inter-ICC	0.76	0.70	0.83	0.58	0.66	0.84	0.89	0.84	0.80
SPGR T1rho	Intra-ICC	0.83	0.77	0.70	0.81	0.73	0.87	0.89	0.86	0.83
	Inter-ICC	0.58	0.44	0.44	0.46	0.41	0.70	0.79	0.63	0.62
T2	Intra-ICC	0.95	0.93	0.96	0.92	0.93	0.96	0.95	0.92	0.96
	Inter-ICC	0.72	0.64	0.66	0.58	0.68	0.75	0.63	0.70	0.75

Table 6 shows intra- and inter-ICC of relaxation time of each ROI in vertical analysis. Intra-ICC of T1rho and T2 from total LM and MM were excellent. In the analysis of each segment, intra-ICC of the medial and lateral meniscal segments on b-FFE were excellent (0.93–0.95), while those on SPGR were lower than those on b-FFE and were good to excellent (0.73–0.87 in the LM and 0.90–0.92 in the MM). Intra-ICC of each segment on T2 were all excellent (0.95–0.98). Inter-ICC from total LM and MM were excellent on b-FFE and T2, and good on SPGR. Each segmental analysis was good to excellent (0.69–0.91) on b-FFE, good to excellent (0.60–0.78) on SPGR, and excellent (0.77–0.93) on T2. On T1rho, inter-ICC on b-FFE were higher than those on SPGR.

**Table 6:** Intra- and inter-class correlation coefficient (intra- and inter-ICC) in vertical zonal analysis

		total LM and MM	Lateral meniscus		Medial meniscus	
			LMAH	LMPH	MMAH	MMPH
b-FFE T1rho	Intra-ICC	0.94	0.95	0.94	0.93	0.95
	Inter-ICC	0.81	0.69	0.86	0.82	0.91
SPGR T1rho	Intra-ICC	0.86	0.87	0.73	0.90	0.92
	Inter-ICC	0.68	0.67	0.60	0.63	0.78
T2	Intra-ICC	0.97	0.95	0.96	0.96	0.98
	Inter-ICC	0.88	0.77	0.90	0.85	0.93

## Discussion

There have been prior studies which describe differences in collagen and proteoglycan content in different meniscal zones. One study used electron microscopy to reveal three distinct meniscal layers: 1) superficial network: the tibial and femoral surfaces are covered by a meshwork of thin fibrils, 2) lamellar layer: a layer of lamellae of collagen fibrils beneath the superficial network, and 3) central main layer: bundles of collagen fibrils oriented in a circular manner [17]. A study using hematoxylin and eosin staining and immunohistochemical staining revealed vertical zonal differentiation: fibrocartilage without any vessels was demonstrated in the white zone and highly vascularized loose and areolar connective tissue was demonstrated in the red zone [18]. There have been several animal studies describing meniscal zonal contents. In an experiment with bovine subjects, the inner one-third of meniscus is composed of 60% type II and 40% type I collagen, while the predominant collagen of the outer two-thirds is type I with a trace amount of types III and V [19]. In a study with procine, collagen content in the central zone was lower than in the peripheral zone, and glycosaminoglycan content in the central zone was higher than that in the peripheral zone [20]. The surfaces of the femur and tibia did not contain glycosaminoglycans [20].

Several studies have described the correlation between T1rho and T2 relaxation times with collagen or proteoglycan composition in articular cartilage. Akella et al. reported that an increase in T1rho relaxation time has a strong correlation with loss of proteoglycan in articular cartilage [21]. Goodwin et al. reported that T2 relaxation time is related to collagen fiber orientation in articular cartilage [22]. Since the extracellular matrix of meniscus is composed mainly of water, collagen and proteoglycans (similar to articular cartilage), we can expand the same discussion about relaxation times in articular cartilage to the meniscus. In other words, higher T1rho relaxation time likely represents relatively lower proteoglycan content, and differences in T2 relaxation time may represent differences of water content and collagen fiber orientation in the meniscus.

In our study, there were zonal differences in T1rho and T2 relaxation times of adult meniscus without symptom and imaging abnormality. In most segments, T1rho and T2 relaxation times of the superficial zone were significantly higher than those of the deep zone. Likewise, T1rho and T2 relaxation times of the inner zone were significantly higher than those of the outer zone. Higher T1rho relaxation time in the superficial zone and inner zone may represent relatively lower proteoglycan content. Higher T2 relaxation time in these zones may represent differences in collagen fiber orientation and/or increase in water.

There have been a few studies about vertical zonal differentiation of MR relaxation times of normal meniscus. Calixto et al. reported that, for both osteoarthritis and control subjects, the MMPH and LMPH showed significantly higher T1rho and T2 relaxation time in the inner zone compared to the middle and outer zones [15]. They suggested that different patterns of zonal T1rho and T2 relaxation times were caused by differences in macromolecular organization such as collagen ultrastructure and possible collagen cross linking [15]. Our results of T1rho comparison between inner and middle zones in MMAH and MMPH are consistent with Calixto’s findings. Similarly, our results of T2 comparison between inner and middle zones in LMAH and MMPH are consistent with Calixto’s findings. On the other hand, Tsai et al. reported that the T2 relaxation time increased significantly from the inner zone to the outer zone of LMPH and MMPH [7]. They suggested that the highest T2 value of the outer zone reflected the abundant blood vessels within vascular zone, and lowest relaxation time of the inner zone was related to the main substance of circumferential fibers [7]. Our result is consistent to Tsai’s study in LMPH, but not in MMPH. This discrepancy in MMPH may be due to differences in the methods of vertical meniscus segmentation. In vertical segmentation, we included the layer of superficial zone which was not included in Tsai’s study. In our study, since T2 relaxation time of the superficial zone was higher than that of the deep zone, relatively larger proportions of superficial zone in our inner zone may contribute to higher average T2 relaxation times of the inner zone. In addition, the degree of vascularity in each subject may influence the results, especially related to age, although the mean age of the subjects in our study was similar to Tsai’s study (29 years vs. 26.5 years, respectively) [7].

To our knowledge, no studies have been published to date about horizontal zonal analysis of meniscal T1rho and T2 relaxation times. Horizontal zonal difference inT1rho relaxation time of the meniscus may represent the differences of proteoglycan composition which contributes to strength against compressive stress, since compressive stress in the superficial zone of the meniscus is greater than in the deep zone. This can be explained by the same mechanism and depth dependent T1rho changes of articular cartilage of the knee. Nozaki et al. reported that average T1rho values in the superficial layer of the femoral cartilage were higher than those in the deep layer in the most locations of the healthy subjects’ femoral condyles [23]. Horizontal zonal difference of T2 relaxation time of the meniscus may also be explained similarly to articular cartilage, which demonstrates higher T2 relaxation time in the superficial zone than in the deep zone. Kaneko et al. reported that T2 relaxation time in the superficial zone of healthy articular cartilage was higher than in the deep zone [24].

The results in this study show there are zonal variations in menisci with no signal abnormalities on conventional MRI sequence in asymptomatic knees. Our results will be good reference in quantitative analysis for detecting slight meniscus injury or early stage of meniscus degeneration. Further studies with histological analysis would be needed to confirm our findings.

Compared with SPGR, b-FFE T1 rho sequence showed more significant zonal differentiation with higher inter- and intra-observer reproducibility. The reason for this result may be that the advantages of b-FFE sequences include higher signal-to-noise ratio (SNR) and better contrast-to-noise ratio (CNR) as compared with SPGR sequences [25]. In our study, average T1rho relaxation times of the meniscus on SPGR sequence were higher among all zones than b-FFE sequence. However, Nozaki et al. reported that the average T1rho value of the entire femoral cartilage with b-FFE was significantly higher compared to SPGR, and they discussed that the difference may be due to difference in sensitivity to proteoglycan content between the two sequences [26].

There are some limitations to our study. First, the number of subjects was small. Second, subjects recruited for this study had no confirmation with arthroscopy or surgery that there was no meniscus injury or degeneration. However, no subjects in this study had any abnormalities of shape or signal intensity in the meniscus with proton density weighted MR imaging. Third, we have no histological or immunohistochemical correlation. Fourth, in this study, average T1rho relaxation times of the meniscus were between 72.0 ms and 97.2 ms on b-FFE T1rho, and 80.7 ms and 104.9 ms on SPGR T1rho in horizontal or vertical zonal analysis. Average T2 relaxation times were between 60.3 ms and 104.6 ms. These values were higher than other reports of T1rho or T2 relaxation times of healthy meniscus [6, 7, 10, 13, 15]. T1rho and T2 relaxation times may vary with different imaging sequences and vendors. However, some pixels in the ROIs were eliminated for MR relaxation time analysis in the present study because calculated relaxation time of these pixels are 0 due to misregistration or inadequate fitting. These pixels were more prominent on SPGR T1rho and T2 relaxation time sequence. This can be one of the reasons relaxation times of SPGR T1rho relaxation times are higher than those of b-FFE T1rho in the present study. This fact may affect the results of zonal MR relaxation time analysis. To address this problem, higher signal-to-noise sequences including TSL = 0 for T1rho mapping and shorter TE for T2 mapping may be obtained.

## Conclusions

There were zonal differences in relaxation times of the normal meniscus. In most segments, T1rho and T2 relaxation times of superficial zone were higher than those of deep zone, and T1rho and T2 relaxation times of inner zone were higher than those of outer zone. These differences can be due to zonal variations of collagen and glycosaminoglycan composition, although this speculation needs to be confirmed with histological or immunohistochemical analysis. Compared with SPGR T1rho, b-FFE T1rho showed more significant zonal differentiation with higher inter- and intra-observer reproducibility.

## Abbreviations

b-FFE: balanced-fast field echo
DICOM: Digital Imaging and Communications in Medicine
inter-ICC: inter-class correlation coefficients
intra-ICC: intra-class correlation coefficients
LM: Lateral meniscus
LMAH: Lateral meniscus anterior horn
LMBD: Lateral meniscus body
LMPH: Lateral meniscus posterior horn
MIPAV: Medical image processing, analysis and visualization
MM: Medial meniscus
MMAH: Medial meniscus anterior horn
MMBD: Medial meniscus body
MMPH: Medial meniscus posterior horn
ProSet: Principle of selective excitation technique
ROI: Region of interest
SPGR: Spoiled gradient-recalled acquisition
SPIR: Spectral presaturation with inversion recovery
T1rho: T1 relaxation time in the rotating frame
